# Supplementary material for: Effectiveness and Safety of Ayurvedic Medicines in Type 2 Diabetes Mellitus Management: A Systematic Review and Meta-Analysis
Source: Front Pharmacol. 2022 Jun 8;13:821810. doi: 10.3389/fphar.2022.821810 (PMC9213670; doi:10.3389/fphar.2022.821810)
Supplement: Supplementary file 1 [file DataSheet1.zip › Supplementary Material/Forest and Funnel Plots/Enicostemma axillare (versus OAD)/FBG.pdf]

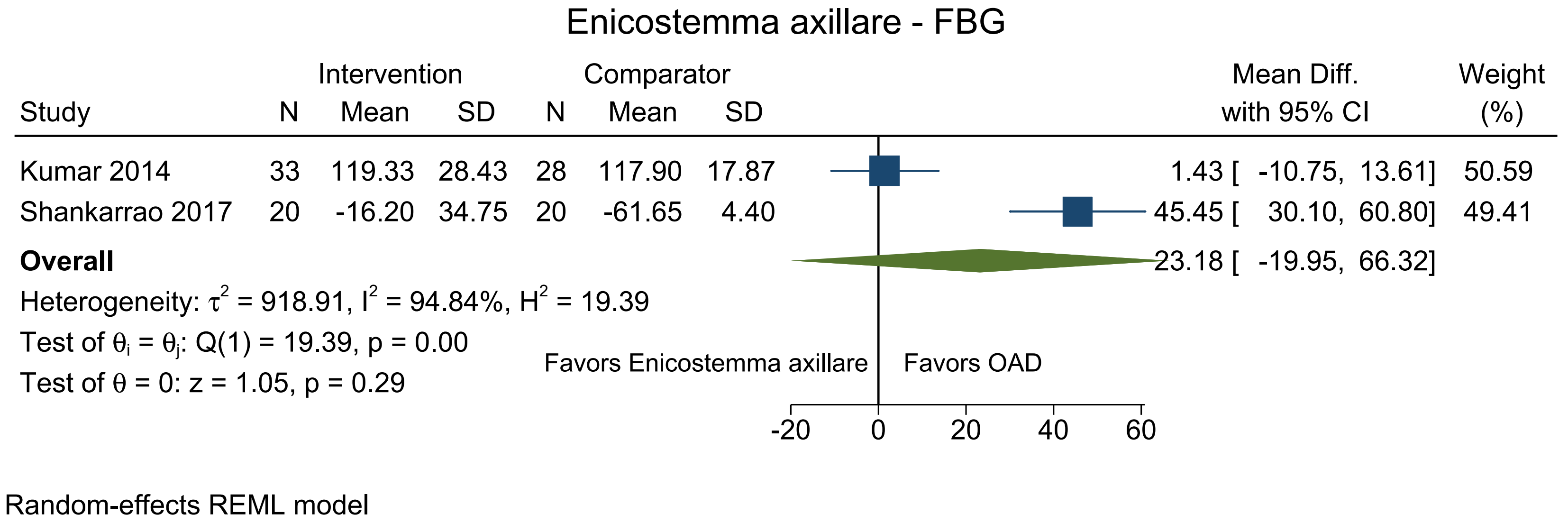

Enicostemma axillare - FBG
Intervention
Comparator
Study N Mean SD N Mean SD
Mean Diff. with 95% CI
Weight (%)
Kumar 2014 33 119.33 28.43 28 117.90 17.87 1.43 [-10.75, 13.61] 50.59
Shankarrao 2017 20 -16.20 34.75 20 -61.65 4.40 45.45 [30.10, 60.80] 49.41
Overall 23.18 [-19.95, 66.32]
Heterogeneity: τ² = 918.91, I² = 94.84%, H² = 19.39
Test of θi = θj: Q(1) = 19.39, p = 0.00
Test of θ = 0: z = 1.05, p = 0.29
Favors Enicostemma axillare
Favors OAD
-20 0 20 40 60
Random-effects REML model
